# Improved Outcomes Associated With Teduglutide Use in Patients With Both Short Bowel Syndrome and Crohn’s Disease

**DOI:** 10.1093/crocol/otae007

**Published:** 2024-01-24

**Authors:** Rex K Siu, Christian Karime, Jana G Hashash, Jami Kinnucan, Michael F Picco, Francis A Farraye

**Affiliations:** Department of Medicine, Mayo Clinic, Jacksonville, FL, USA; Department of Medicine, Mayo Clinic, Jacksonville, FL, USA; Inflammatory Bowel Disease Center, Department of Gastroenterology and Hepatology, Mayo Clinic, Jacksonville, FL, USA; Inflammatory Bowel Disease Center, Department of Gastroenterology and Hepatology, Mayo Clinic, Jacksonville, FL, USA; Inflammatory Bowel Disease Center, Department of Gastroenterology and Hepatology, Mayo Clinic, Jacksonville, FL, USA; Inflammatory Bowel Disease Center, Department of Gastroenterology and Hepatology, Mayo Clinic, Jacksonville, FL, USA

**Keywords:** Crohn’s disease, teduglutide, glucagon-like peptide 2 agonist, parenteral support, short bowel syndrome, intestinal failure, diarrhea

## Abstract

**Introduction:**

Crohn’s disease (CD) with short bowel syndrome (SBS) can present as chronic intestinal failure (CIF) often requiring nutritional support. Teduglutide is a treatment option for these patients. We investigated clinical outcomes of CD-CIF patients with SBS treated with teduglutide.

**Methods:**

Adults with CD-CIF and SBS who received teduglutide were identified at a tertiary care academic center between 2012 and 2023. Data was collected retrospectively. Primary outcome measured was reduction in parenteral support (PS) by ≥20% volume, with PS defined as utilization of parenteral nutrition (PN) or intravenous fluids (IVF). Several secondary outcomes included immunosuppressive medication changes, subjective symptom improvement, and stool output.

**Results:**

We identified 32 patients with CD-CIF and SBS receiving teduglutide. Comparing clinical outcomes before and after teduglutide, 26 of 32 patients achieved the primary outcome of ≥20% PS reduction. A decrease was seen in patients requiring PN + IVF, with corresponding increases in patients requiring PN only and IVF only. Among all 3 groups, a total of 23 patients received PN prior to teduglutide, which decreased to 14 following teduglutide. Weekly PN volume reduced from 7.00 to 3.55 L and weekly frequency decreased from 7.00 to 3.00 instances (*P* < .01). Reductions in weekly volume and frequency were observed among all patients receiving IVF support (25 vs 15). Secondary outcomes showed improvement in patient reported subjective symptoms (84.4%), stool output (90.6%), patients meeting criteria for diarrhea/high ostomy output (27 vs 14), and use of unique antidiarrheal medications (3.0 vs 2.0).

**Conclusions:**

This retrospective case series demonstrated improved clinical outcomes in patients with CD-CIF and SBS treated with teduglutide resulting in decreased PS requirements, antidiarrheal medications requirement, and stool output without significant effects on immunosuppressive therapy.

## Graphical Abstract

**Graphical Abstract:**
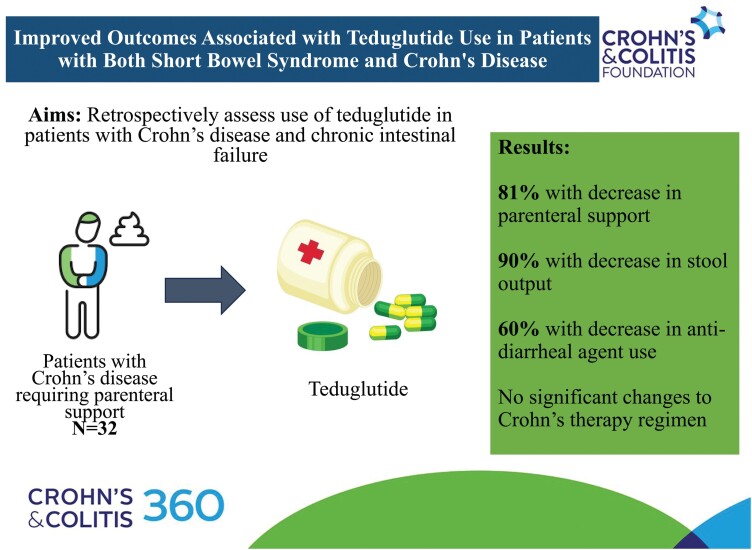

## Introduction

Thought to be caused by immune dysregulation in a susceptible host, inflammatory bowel disease (IBD) affects 1 in 209 adults in the United States with a worldwide prevalence exceeding 0.3%.^1,2^ In patients with IBD, those with Crohn’s disease (CD) may develop chronic intestinal failure (CIF), defined by the European Society for Clinical Nutrition and Metabolism (ESPEN) as a “reduction of gut function below the minimum necessary for absorption of macronutrients and/or water and electrolytes, such that intravenous supplementation is required to maintain health and/or growth.”^3^ CIF may occur secondary to complications from abdominal surgery, repeated intestinal resections resulting in short bowel syndrome (SBS), or primary CD activity.^4,5^

A study conducted in a United Kingdom registry found that among patients with CIF, CD was the underlying disease in 32% of patients.^6^ Additionally, a large Japanese study of 1703 patients with CD by Watanabe et al. noted a 20-year cumulative risk of 8.5% for CIF following initial surgery.^7^ Patients with CIF often develop malnutrition, requiring parenteral support (PS) in the form of parenteral nutrition (PN) and/or intravenous fluids (IVF) with electrolytes. Besides PS reliance resulting in considerable lifestyle and psychosocial stressors in patients with CIF, PS is often costly with an estimated median annual healthcare charge of 3427 United States Dollars (USD) in patients without IBD and 51 456 USD in patients with IBD.^8^

Teduglutide, a glucagon-like peptide 2 agonist (GLP2), has been used in the treatment of patients suffering from SBS with chronic intestinal failure (SBS-CIF) requiring PS, including patients with CD and CIF (CD-CIF). Previous studies have shown that use of teduglutide may reduce the volume of PS requirement in patients with CD-CIF.^9–12^ However, studies on patients with CD-CIF have been limited to case reports,^13–16^ cohort studies with 15 or fewer CD patients,^10,12^ and one study of 18 SBS-CIF patients, which included 5 with CD-CIF.^11^ Although these studies lend insight into use of teduglutide in patients with CD requiring varying degrees of nutritional support, outcomes investigated have been limited to reduction in PS and burden of healthcare costs given the small number of patients. Given current limited literature, we sought to expand the available data on use of teduglutide in patients with CD through retrospective review at a large tertiary care referral center. Our primary outcome of interest was reduction in PS by ≥20% volume. Secondary outcomes included patient-reported symptomatic improvement, volume of stool output, use of antidiarrheal medications, immunosuppressive medication regimen, and incidence of hospital care utilization.

## Methods and Materials

### Patient Selection

We retrospectively identified adult patients with a diagnosis of CD and SBS receiving teduglutide therapy at our tertiary care academic center between January 2012 and January 2023. The time period of 2012–2023 was selected given initial United States Food and Drug Administration approval of teduglutide in 2012. Our initial cohort of patients was identified using ICD-10 codes for CD (K50.*) and SBS (DA96.*), with manual verification of teduglutide therapy through medication documentation and refill requests. Verification of CD diagnosis (confirmed by biopsy) and SBS (by documentation or imaging/surgical findings nothing less than 150 cm of remaining small bowel) was performed.

**Figure 1.**
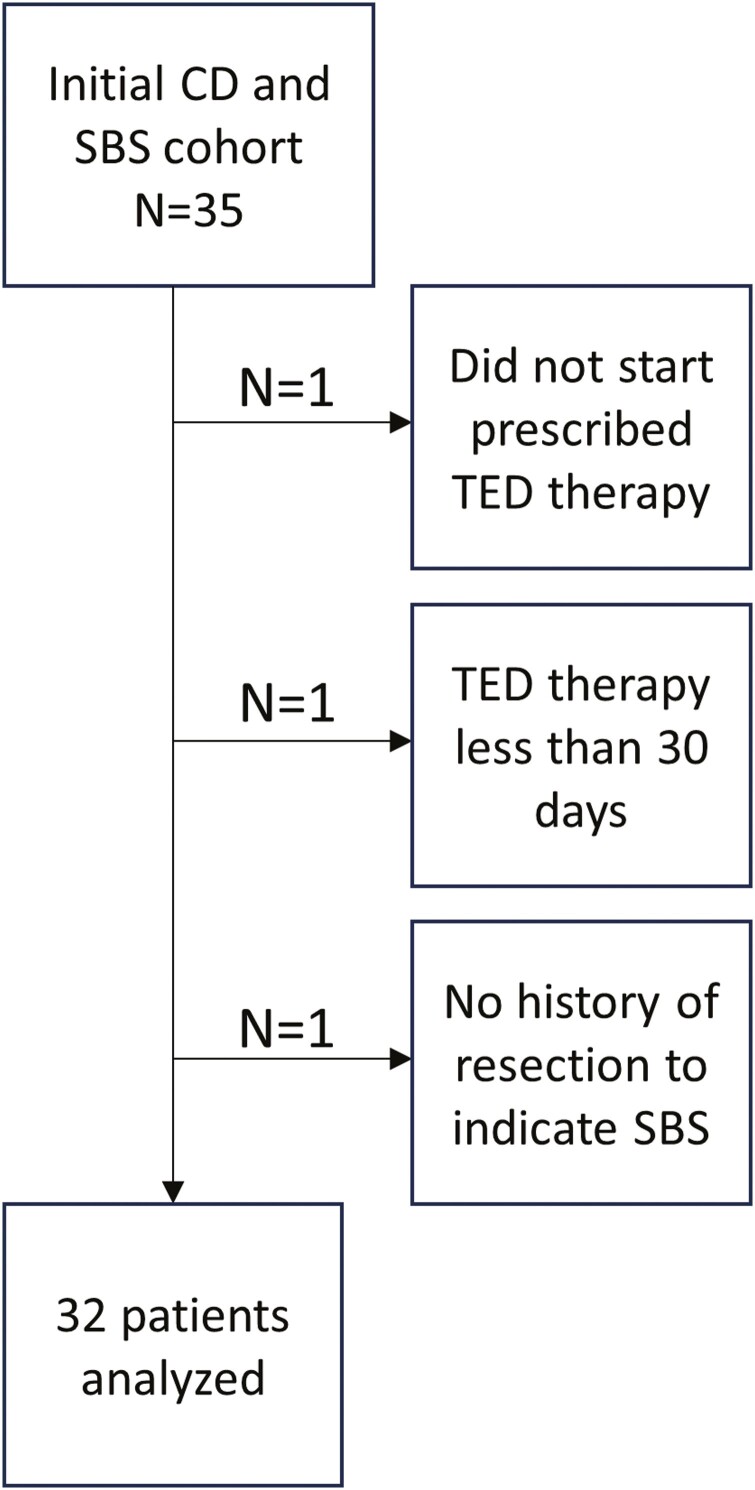
Patient flow diagram.

An initial cohort of 35 patients with CD and SBS on teduglutide therapy was identified. Subsequent exclusion of patients based on teduglutide therapy less than 30 days (*n* = 2) and lack of documented SBS per surgical resection documentation (*n* = 1) was performed, with a final cohort of 32 patients as shown in Figure 1. IRB approval was obtained to collect deidentified patient information.

### Data Collection

Baseline patient characteristics were obtained, including age, sex, ethnicity, BMI, comorbid conditions, Charlson Comorbidity Index (CCI), previous gastrointestinal surgeries performed, and CD-specific clinical characteristics according to Montreal classification (age at diagnosis, disease location, disease phenotype). IBD-specific immunosuppressive therapy was also classified, with patients categorized by number of immunosuppressive medications. Immunosuppressive medications were defined as tumor necrosis factor alpha antagonists, integrin antagonists, interleukin antagonists, janus kinase antagonists, thiopurines, and methotrexate.

Clinical outcomes were compared before versus after teduglutide therapy. Our primary outcome was reduction of PS volume by ≥20%. Given that PS needs and compositions may vary, PS was further categorized into 3 groups; PN + IVF, PN alone, or IVF alone to delineate nutritional support needs and fluid support needs (malnutrition vs dehydration). PS needs was extracted via clinical documentation of PN formulas and IVF when the patient was in an outpatient setting.

Secondary outcomes included antidiarrheal medication use (defined as documentation of antidiarrheal agent usage and active prescriptions as verified by chart review), subjective patient-reported symptomatic improvement, hospital and emergency care utilization, and stool output meeting definition for diarrhea (≥3 bowel movements per day or ≥1.5 L per day ostomy output). Subjective symptomatic improvement was defined by documentation of patient-reported improvement in symptomatology as documented in the electronic medical records, including energy level, quality of life, or bowel output following teduglutide initiation. Incidence of emergency room utilization and hospitalization for diagnoses of acute kidney injury or abdominal pain per 100 days of teduglutide therapy was also characterized.

### Statistical Analysis

Descriptive statistics were utilized to summarize baseline characteristics as medians with interquartile ranges (IQR) for continuous variables, and proportions for categorical variables. Wilcoxon Rank Sum and Fisher Exact tests were performed to compare outcomes prior to and after initiation of teduglutide therapy. A *P* value of <.05 was used to denote statistical significance. Statistical analysis was performed using SPSS statistical software (version 25, IBM SPSS).

## Results

### Patient Characteristics

We identified 32 patients with CD and SBS receiving teduglutide therapy in our analysis. Patient demographics are detailed in Table 1. Median age at teduglutide initiation was 60.6 years (IQR: 48.4–68.3 years) with a median therapy duration of 782.5 days (IQR: 386–1600 days). The most common comorbid conditions were chronic kidney disease (28.1%), type 2 diabetes (18.8%), and chronic obstructive pulmonary disease (12.5%). Median Charlson Comorbidity index was noted at 3.0 (IQR: 1.0–5.0). CD Montreal classification is summarized in Table 1, with most common disease location being ileocolonic (90.6%) and phenotype being fistulizing (65.6%). All patients had undergone ≥1 gastrointestinal surgeries prior to initiating teduglutide therapy, the most common of which were small bowel resection (78.8%), total colectomy (50%), and partial colectomy (34.4%). Of the 32 patients, 71.9% had a history of an ileostomy and 6.3% of a jejunostomy, with 19 patients (59.3%) having an active ostomy at initiation of teduglutide therapy.

**Table 1.** Baseline characteristics.

	Median (IQR) or fraction (%)
Variable	All patients *N* = 32
*Demographics*	
Age at teduglutide initiation (years)	60.6 (48.4–68.3)
Duration of teduglutide therapy (days)	781.5 (385.5–1599.5)
Male	11 (34.4%)
Caucasian	30 (93.8%)
*Comorbid conditions prior to teduglutide initiation*	
Inflammatory bowel disease	
Crohn’s disease	32 (100.0%)
Diabetes mellitus	6 (18.8%)
Chronic obstructive pulmonary disease	4 (12.5%)
Chronic kidney disease	9 (28.1%)
Congestive heart failure	1 (3.1%)
Liver cirrhosis	1 (3.1%)
Transplant (any)	1 (3.1%)
Autoimmune disease (any)	3 (9.4%)
Charlson Comorbidity Index	3.0 (1.0–5.0)
BMI	21.9 (19.2–25.5)
Gastrointestinal surgery (not mutually exclusive)	32 (100%)
Total colectomy	16 (50.0%)
Partial colectomy	11 (34.4%)
Small bowel resection	26 (78.8%)
Ileostomy	23 (71.9%)
Colostomy	0 (0%)
Jejunostomy	2 (6.3%)
Ostomy at time of therapy	19 (59.3%)
Re-anastomosis	10 (31.3%)
Loss of ileocecal valve	32 (100%)
*Montreal classification*	
Montreal—Age of onset	
A1: ≤16 years	4 (12.5%)
A2: 17–39 years	23 (71.9%)
A3: ≥40 years	5 (15.6%)
Montreal—Disease location	
L1: Ileal	2 (6.3%)
L2: Colonic	1 (3.1%)
L3: Ileocolonic	29 (90.6%)
L4: Isolated upper	0 (0.0%)
Montreal—Disease phenotype	
B1: Non-stricturing, non-penetrating	4 (12.5%)
B2: Stricturing	7 (21.9%)
B3: Penetrating/fistulizing	21 (65.6%)
p: Perianal disease	18 (56.3%)

### Parenteral Support

Distribution of patients among the 3 groups (PN + IVF, PN only, and IVF only) prior to initiation of teduglutide therapy showed that 16 (50%) patients required PN + IVF, 7 (21.9%) required PN only, and 9 (28.1%) required IVF only (Table 2). When looking at this in relation to our primary outcome of ≥20% volume reduction of PS (PN and/or IVF) following teduglutide initiation, 6 patients (18.8%) noted a PS reduction of <20% whilst another 6 patients (18.8%) had a 20%–50% reduction in PS. A PS reduction of 51%–80% occurred in 12 patients (37.5%), whilst 8 patients had >80% reduction in PS volume. All 8 patients experiencing >80% reduction achieved complete parenteral independence. In these 8 patients, 2 previously required PN + IVF, 2 previously required PN only, and 4 previously required IVF only.

**Table 2.** Primary outcomes.

	Median (IQR) or fraction (%)		
Parenteral support^a^ (PS)	Before teduglutide (*N* = 32)	After teduglutide (*N* = 32)	*P* value
Parenteral nutrition and intravenous fluids (PN + IVF)	16/32 (50%)	5/32 (15.6%)	<.01
Switched to PN only	—	4/16 (25.0%)	NA
Switched to IVF only	—	5/16 (31.3%)	NA
Parenteral nutrition only (PN)	7/32 (21.9%)	9/32 (28.1%)	.16
Intravenous fluids only (IVF)	9/32 (28.1%)	10/32 (31.3%)	.32
No longer requiring PN or IVF	0/32 (0%)	8/32 (25.0%)	<.01
Previously on PN + IVF	—	2/8 (25.0%)	NA
Previously on PN only	—	2/8 (25.0%)	NA
Previously on IVF only	—	4/8 (50.0%)	NA
*Volume and frequency of PS* ^a^			
Patients on PN	23/32 (71.8%)	14/32 (43.8%)	<.01
Volume per daily infusion (L)	1.60 (1.25–2.08)	1.35 (1.03–1.90)	<.01
Volume per week (L)	7.00 (3.0–7.0)	3.55 (0.0–7.0)	<.01
Frequency per week (#)	7.00 (3.9–7.0)	3.00 (0.0–7.0)	<.01
Patients on IVF	25/32 (78.1%)	15/32 (46.9%)	<.01
Volume per infusion	1.00 (1.00–1.00)	1.00 (1.00–1.00)	1.00
Volume per week	7.00 (3.00–7.00)	3.00 (0.00–7.00)	<.01
Frequency per week	7.0 (3.0–7.0)	3.0 (0.0–7.0)	<.01
*Decrease in PS needs*			
Decrease by < 20% weekly volume	—	6/32 (18.75%)	NA
Decrease by 20%–50% weekly volume	—	6/32 (18.75%)	NA
Decrease by 51%–80% weekly volume	—	12/32 (37.5%)	NA
Decrease by >80% weekly volume	—	8/32 (25%)	NA

^a^Parenteral support defined as receiving intravenous nutrition and/or hydration with percent decrease in weekly volume reflecting the combined volume of parenteral nutrition and parenteral fluids.

Of the 16 patients in the PN + IVF group, only 5 patients (31.3%) continued to need PN + IVF following therapy, with 11 patients (68.8%) able to deescalate parenteral needs. Of the 11 patients who were able to deescalate parenteral needs, 5 stopped PN, 4 stopped IVF, and 2 no longer required either. Those changes resulted in increases in patients requiring PN only (from 7 to 9 patients) and IVF only (from 9 to 10 patients). Assessing patients among all 3 groups, 23 patients were receiving PN prior to teduglutide therapy; 16 from the IVF + PN group and 7 from the PN-only group (Table 2). Following therapy, PN-dependent patients decreased to 14 (71.9% vs 43.8%, *P* < .01), with reduction in weekly PN frequency (7.0 times vs 3.0 times per week, *P* < .01), infusion amount per session (1.6 vs 1.35 L, *P* < .01), and total infusion amount per week (7.0 vs 3.5 L, *P* < .01). When comparing patients receiving IVF prior to (*n* = 25) and after (*n* = 15) teduglutide therapy, a significant reduction in IVF frequency per week (7.0 times vs 3.0 times per week, *P* < .01) and total infusion amount per week (7.00 vs 3.00 L, *P* < .01) was noted as well.

### Stool Output, Symptoms, and Antidiarrheal Medication Use

Of 32 patients, 27 met the definition of diarrhea (≥3 bowel movements per day or ≥1.5 L per day ostomy output) prior to teduglutide therapy. Following teduglutide therapy, this number was reduced to 14 patients (84.4% vs 43.8%, *P* < .01, Table 3). In terms of antidiarrheal medication use prior to teduglutide therapy, 90.6% of patients utilized one or more antidiarrheal medications to control stool output, with a median use of 3.0 (IQR 2.0–3.0) unique antidiarrheal medications (Table 4). Following teduglutide therapy, a nonsignificant decrease in patients utilizing antidiarrheal medications was noted (90.6% vs 84.4%, *P* = .16), although a statistically significant decrease was noted in the median number of antidiarrheal medications used (3.0 vs 2.0, *P* < .01). An increase in the number of patients using one or less antidiarrheal medications was also noted (15.6% vs 46.9%, *P* < .01). In terms of individual antidiarrheal medications used, a reduction was noted in the use of loperamide (84.4% vs 59.4%, *P* < .01), diphenoxylate-atropine (87.5% vs 59.4%, *P* < .01), and tincture of opium (43.8% vs 12.5%, *P* < .01). No difference was noted in the use of pancreatolipase (18.8% vs 15.6%, *P* = .32) or octreotide (9.4% vs 3.1%, *P* = .16). On subjective assessment, clinical documentation revealed 90.6% of patients reported stool output decreasing by ≥30% when looking at reported frequency and volume of ostomy output, whilst 84.4% reported meaningful improvement in symptoms including increased energy levels, decreased stool frequency and output, or improved mood following teduglutide initiation (Table 3).

**Table 3.** Secondary outcomes.

	Median (IQR) or Fraction (%)		
Variable	Before teduglutide (*N* = 32)	After teduglutide (*N* = 32)	*P* value
Stool output and symptom improvement			
Stool output meeting definition of diarrhea (ostomy ≥1.5 L per day or ≥3 bowel movements per day)	27/32 (84.4%)	14/32 (43.8%)	<.01
Stool output decrease by ≥30%	—	29/32 (90.6%)	NA
Meaningful subjective symptom improvement	—	27/32 (84.4%)	NA
Immunosuppressive medications			
No immunosuppressive med	14/32 (43.75%)	16/32 (50.0%)	.48
One immunosuppressive med	16/32 (50.0%)	15/35 (46.9%)	.74
Two or more immunosuppressive med	2/32 (6.25%)	1/32 (3.1%)	.16
Change in immunosuppressant medication use			
Change (any)	—	10/32 (31.3%)	NA
Immunosuppressive med escalation	—	4/32 (12.5%)	NA
Immunosuppressive med deescalation	—	6/32 (18.8%)	NA
Healthcare utilization			
Emergency department visits for acute kidney injury or abdominal pain (per 100 days on therapy)	0.07 (0.00–0.24)	0.08 (0.00–0.31)	.94
Hospitalization for acute kidney injury or abdominal pain (per 100 days on therapy)	0.00 (0.00–0.17)	0.00 (0.00–0.23)	.97

**Table 4.** Antidiarrheal agents.

	Median (IQR) or Fraction (%)		
Variable	Before teduglutide (*N* = 32)	After teduglutide (*N* = 32)	*P* value
Receiving medications for stool output control	29/32 (90.6%)	27/32 (84.4%)	.16
Individual medications (number)	3.0 (2.0–3.0)	2.0 (1.0–2.8)	<.01
One or less medications	5/32 (15.6%)	15/32 (46.9%)	<.01
Loperamide	27/32 (84.4%)	19/32 (59.4%)	<.01
Diphenoxylate - atropine	28/32 (87.5%)	19/32 (59.4%)	<.01
Pancrelipase	6/32 (18.8%)	5/32 (15.6%)	.32
Tincture of opium	14/32 (43.8%)	4/32 (12.5%)	<.01
Octreotide	3/32 (9.4%)	1/32 (3.1%)	.16

### Immunosuppressive Medication Use

Within this cohort, 43.8% of patients were not on immunosuppressive therapy, 50% of patients were on one immunosuppressive agent, and 6.2% of patients were on two or more immunosuppressive agents. No statistically significant difference was noted after teduglutide initiation when characterized in the most recent documentation regarding immunosuppressive therapy, with 50% of patients on no immunosuppressive therapy, 46.9% on one immunosuppressive agent, and 3.1% were on two or more immunosuppressive agents (Table 3). Of the 32 patients, 10 (31.3%) patients underwent a change in immunosuppressive therapy (requiring more agents, less agents, or change in agent class) with 6 patients undergoing deescalation of therapy (requiring at least one less agent) and 4 patients undergoing escalation (requiring at least one more agent).

### Teduglutide Discontinuation and Adverse Events

Of 32 patients, 12 patients (37.5%) discontinued teduglutide due to medication intolerance or financial reasons. Table 5 details reasons for teduglutide discontinuation, with the most common reasons for medication intolerance being abdominal pain, bloating, and discomfort. Additional reasons for teduglutide discontinuation were due to financial or insurance concerns, lack of effectiveness, or planned abdominal surgery (Table 5).

**Table 5.** Discontinued teduglutide and adverse events.

	Fraction (%)
Variable	After teduglutide (*N* = 12)
Discontinuation due to adverse events (*N* = 5)	
Nausea	1/5 (20%)
Abdominal pain	1/5 (20%)
Abdominal bloating	1/5 (20%)
Dysphagia	1/5 (20%)
Gastrointestinal bleeding	1/5 (20%)
Discontinuation not associated with adverse events (*N* = 7)	
Insurance or cost limitation	3/7 (42.8%)
Impending surgery	1/7 (14.3%)
No improvement on therapy	2/7 (28.5%)
After achieving enteral autonomy	1/7 (14.3%)

## Discussion

### Available Literature

CD-CIF has limited treatment options, but one of the available therapies is the glucagon-like peptide analog teduglutide. Since teduglutide was approved for FDA use in the United States in 2012, there have been few post-market cohort studies looking into its long-term effects on clinical outcomes. Prior studies by Kochar et al., Puello et al., and Sato et al. looking at SBS-CIF and CD-CIF patients requiring PS show teduglutide’s ability to significantly reduce PS.^10–12^ The current study adds to the limited body of literature on teduglutide use in patients with CD, reporting on the clinical variables of PS requirement, symptomatic improvement, stool output, and use of antidiarrheal medications. Given that prior studies have been limited to fewer than 15 CD-CIF patients, the current study represents the largest reported study to date and significantly adds to the limited clinical literature on the topic.

In regards to our primary outcomes of reduction in PS requirement by ≥20% volume, the current study demonstrated a statistically significant decrease in percent weekly volume of PS in patients with CD-CIF following teduglutide initiation, with further delineation of PS between 3 groups of PN + IVF, PN only, and IVF only. Additionally, following teduglutide initiation, patients with CD-CIF reported improved symptomatology and reduction in stool output. Furthermore, most patients experienced reduction in antidiarrheal medication use, further supporting the notion of clinical improvement of CIF in patients with CD following teduglutide therapy initiation. The findings related to PS reduction are congruent with prior reports; however, our study augments prior reports by indicating that teduglutide therapy can reduce the need for antidiarrheal medications and provide significant reductions in stool output without having significant effects on CD immunosuppressive regimen.

### Glucagon-like Peptide 2 Effects

It has been surmised that CD-CIF patients who have undergone terminal ileal and colonic resection may achieve greater response to teduglutide therapy; this may be due to lower levels of endogenous GLP2 since studies have shown the colon and terminal ileum being the regions of endogenous GLP2 production.^17,18^ This observation may be why the patients in this study experienced significant response to teduglutide therapy, with 84.4% of patients having previously undergone full or partial colectomy (with terminal ileum resection in 100% of our cohort). Another study noted that CD-CIF demonstrates low CD disease recurrence following CIF diagnosis and they observed that immunosuppressive therapy remained stable over the study period.^19^ This is consistent with our observation, as minimal medication changes occurred following teduglutide initiation.

### Limitations

The strength of our study includes expanded outcomes with the largest cohort to date. Nevertheless, limitations are present and as such warrant discussion. Firstly, the nature of our retrospective study design lacked a control group. During study design, it was felt that comparison between patients with CD-CIF severe enough to necessitate teduglutide therapy to patients with CD-CIF not necessitating advanced therapy may result in difficult to interpret results due to nature and burden of disease. As such, our study rather provides a large case series of patient outcomes before versus after teduglutide therapy. Further prospective studies comparing patients with CD-CIF necessitating advanced therapy who are versus are not initiated on teduglutide would be of benefit to further elucidate benefit of teduglutide therapy.

Secondly, our study represents a single-center retrospective study and as such the variables of subjective symptomatic improvement and stool output are abstracted from review of the electronic medical record and documentation of patient-reported symptomatology. As such, an objectively validated scale for the reporting of symptomatic improvement was not available in this retrospective study.

Lastly, given the retrospective abstraction of clinical variables and varying treatment duration, the interval of assessment was set at any clinical documentation prior to teduglutide initiation and at most recent clinical evaluation. As such, data were inconsistent regarding time off PS following therapy. Furthermore, accurate and reliable reporting of IBD disease activity using objective serum measurements or endoscopic data was not consistently available across patients, and as such IBD disease activity prior to and following teduglutide initiation could not be assessed meaningfully.

### Conclusion

While prior studies have investigated reduction in PS and healthcare costs incurred following teduglutide initiation, the current study represents the largest study to date investigating clinical outcomes in patients with CD and SBS on teduglutide therapy. The current study demonstrated that teduglutide initiation in patients with CD-CIF decreased the weekly volume of PS by ≥20% in 81% of the 32 patients, including both PN and IVF. Furthermore, our study indicated a significant symptomatic reduction in over 80% of patients, with 90% noting more than a 30% decrease in stool output. Use of teduglutide was also noted to be associated with reduced need for antidiarrheal medications, including use of loperamide, diphenoxylate-atropine, and tincture of opium. As such, it seems that in patients with CD and SBS initiation of teduglutide may confer both subjective and objective benefits, with reduction in PS volume and alleviation of patient symptoms. Nevertheless, barriers still remain regarding tolerance to adverse effects and financial barriers. Given the predominant Caucasian race of patients in this study, future prospective and multicenter retrospective studies are needed to evaluate whether teduglutide therapy should investigate if racial or gender disparities exist in clinical outcomes given increasing incidence of IBD in non-White populations.^1^

## Data Availability

The data underlying this study was collected from multiple hospitals and is not publicly available. The deidentified data can be provided upon request to the corresponding author.

## Writing Assistance

None declared.
